# Relationship of C5L2 Receptor to Skeletal Muscle Substrate Utilization

**DOI:** 10.1371/journal.pone.0057494

**Published:** 2013-02-27

**Authors:** Christian Roy, Sabina Paglialunga, Gert Schaart, Esther Moonen-Kornips, Ruth C. Meex, Esther Phielix, Joris Hoeks, Matthijs K. C. Hesselink, Katherine Cianflone, Patrick Schrauwen

**Affiliations:** 1 Centre de Recherche Institut Universitaire de Cardiologie & Pneumologie de Québec, Université Laval, Québec, Canada; 2 NUTRIM - School for Nutrition, Toxicology and Metabolism, Department of Human Biology, Maastricht University Medical Center, Maastricht, The Netherlands; 3 NUTRIM - School for Nutrition, Toxicology and Metabolism, Department of Human Movement Sciences, Maastricht University Medical Center, Maastricht, The Netherlands; INSERM/UMR 1048, France

## Abstract

**Objective:**

To investigate the role of Acylation Stimulating Protein (ASP) receptor C5L2 in skeletal muscle fatty acid accumulation and metabolism as well as insulin sensitivity in both mice and human models of diet-induced insulin resistance.

**Design and Methods:**

Male wildtype (WT) and C5L2 knockout (KO) mice were fed a low (LFD) or a high (HFD) fat diet for 10 weeks. Intramyocellular lipid (IMCL) accumulation (by oil red O staining) and beta-oxidation HADH enzyme activity were determined in skeletal muscle. Mitochondria were isolated from hindleg muscles for high-resolution respirometry. Muscle C5L2 protein content was also determined in obese type 2 diabetics and age- and BMI matched men.

**Results:**

IMCL levels were increased by six-fold in C5L2KO-HFD compared to WT-HFD mice (p<0.05) and plasma insulin levels were markedly increased in C5L2KO-HFD mice (twofold, p<0.05). Muscle HADH activity was elevated in C5L2KO-LFD mice (+75%, p<0.001 vs. WT-LFD) and C5L2KO-HFD displayed increased mitochondrial fatty acid oxidative capacity compared to WT-HFD mice (+23%, p<0.05). In human subjects, C5L2 protein content was reduced (−48%, p<0.01) in type 2 diabetic patients when compared to obese controls. Further, exercise training increased C5L2 (+45%, p = 0.0019) and ASP (+80%, p<0.001) in obese insulin-resistant men.

**Conclusion:**

The results suggest that insulin sensitivity may be permissive for coupling of C5L2 levels to lipid storage and utilization.

## Introduction

Acylation Stimulating Protein (ASP; aka C3adesArg) is an adipocyte-derived hormone that promotes triglyceride synthesis in adipose tissue [1]. In humans, there is direct ASP production within the adipose tissue milieu, which increases postprandially and correlates with adipose tissue lipid uptake [2]. Further, fasting ASP levels in humans are predictive of postprandial triglyceride clearance [3]. Fasting plasma ASP is elevated in obesity and insulin resistant states [4], [5]. Based on a physiological role of ASP in humans on enhancing postprandial triglyceride clearance in white adipose tissue (WAT) [3], elevated ASP levels in pathological states (obesity, diabetes) are of interest [6]. It has been proposed that these elevated levels of ASP could further help to stimulate or could be explained as ASP resistance [3], [7].

Most studies have focused on the role of ASP in WAT, where ASP acts via its receptor C5L2 which is expressed in white adipose tissue (WAT) depots [8]. ASP stimulates glucose uptake and triglyceride synthesis in adipocytes and, to a lesser extent, in preadipocytes [9], [10]. Upon ASP stimulation, C5L2 is phosphorylated and internalized [8], initiating a signaling cascade [11], resulting in increased fatty acid (FA) storage and triglyceride synthesis [10], [12], [9]. ASP stimulates fatty acid esterification by increasing diacylglycerolacyltransferase (DGAT) activity which catalyzes the final step in triglyceride synthesis [12]. Furthermore, *in vitro*, ASP supplementation increased lipoprotein lipase (LPL) activity in adipose tissue, facilitating triglyceride accumulation [13], and, consequently, in LPL deficiency, plasma ASP levels are upregulated [14]. ASP also stimulates glucose uptake, inducing translocation of glucose transporters (Glut4 and Glut3) to the cell membrane in cultured human preadipocytes [15].

In addition to WAT, C5L2 is widely expressed in a number of tissues including brain (central nervous system), liver and immune cells [16], [8]. C5L2 is structurally similar to complement factor C5a receptor (C5aR) [17] and also binds C5a and C5adesArg, acting as a decoy receptor for excess C5a under inflammatory conditions of chronic stress such as sepsis and asthma [18], [19].

Glut4 translocation and DGAT activation are also important processes in skeletal muscle, linked with the development of muscle insulin resistance and type 2 diabetes. In that context, it is interesting to note that C5L2 is also expressed in muscle [16]. Similar to adipocytes, ASP increases glucose transport in differentiated L6 muscle myotubes through Glut4 and Glut3 translocation to the cell membrane [15], [20]. However, in contrast to the LPL stimulatory effects in adipocytes, ASP decreases triglyceride accumulation and inhibits LPL-mediated fatty acid uptake and utilization in muscle [13], [21]. Lipid deposits in muscle (intramyocellular lipids, IMCL) are associated with both insulin resistance and enhanced insulin sensitivity according to the athlete’s paradox proposed by Goodpaster [22]. On the other hand, while plasma ASP is increased in states of insulin resistance (as detailed above), plasma ASP decreases following a 2-week training regimen without weight change in young healthy subjects [7], suggesting improved ASP-sensitivity. Studies using C5L2 knockout (C5L2KO) mice have demonstrated both anti-inflammatory [19] and pro-inflammatory [23] roles for C5L2 in mediating immune challenges. From a lipid-glucose metabolism perspective, C5L2KO mice display reduced triglyceride synthesis with absence of ASP response, smaller adipocytes (under high-fat conditions) and delayed postprandial lipid clearance [24]. At the whole-body level, C5L2KO mice are characterized by upregulated fat utilization (reduced respiratory quotient) and high-fat diet induced insulin resistance, suggesting that muscle metabolism may also be affected in C5L2KO mice [24], [25].

While we have shown that C5L2 is a regulator of lipid storage in WAT, the function of C5L2 in muscle remains unknown. In the present study we aimed to address the following question: does whole-body C5L2 deficiency affect skeletal muscle lipid accumulation and substrate utilization? We also examined C5L2 muscle protein expression in obese and type 2 diabetic patients to evaluate a physiological role for C5L2 in man.

## Materials and Methods

### Ethical Statement

The studies involving human participants were approved by the Maastricht University’s institutional medical ethics committee and complied with the principle of the Declaration of Helsinki. All participants were informed of the risks involved in the study and gave their written informed consent. All animal protocols were approved by the Maastricht University and Laval University animal ethics and care committees and were conducted in accordance with their guidelines, and all efforts were made to minimize suffering.

### Mice

Eight-week old male whole-body C5L2KO mice on a C57Bl/6 background, backcrossed at least 10 generations, were generated as previously described [24]. Age-matched C57Bl/6 wildtype (WT) control mice were purchased from Charles River (Montreal, Canada). All mice were housed 2 per cage in a sterile barrier facility with a fade in/fade out 12 h light: 12 h dark cycle (0700 h/1900 h). The cage also contained enrichment toys such as cotton batting and plastic tubes. The mice were placed on either a low-fat diet (LFD; 9% fat, 58% complex carbohydrate (starch) and 33% protein by caloric distribution) (Sniff, Germany) or a high-fat diet (HFD; 60% fat, 20% complex carbohydrate and 20% protein by caloric distribution) (Research Diets, New Brunswick, NJ, USA) for 10 weeks. Body weight was recorded weekly. Prior to dissection, the mice were first sedated (gas mixture of 67% CO_2_ and 33% O_2_), followed by immediate decapitation and blood and tissues were then collected at 0900 h. Hind-leg skeletal muscles were rapidly excised, and placed in ice-cold mitochondrial isolation buffer (see below) while the *tibialis anterior* muscle was frozen in liquid nitrogen-cooled isopentane (2-methyl-butane, Fluka, Zwijndrecht, the Netherlands) and stored at −80°C until further analysis. Blood glucose, plasma triglycerides, non-esterified fatty acids and insulin were measured as previously described [26]. All chemicals were purchased from Sigma Aldrich (St. Louis, MO, USA) unless otherwise noted.

### Muscle Oil Red O Staining and Quantification

Skeletal muscle samples were sectioned into 5 micron thin formaldehyde-fixed cryosections and were incubated with a polyclonal rabbit antibody against basement membrane protein laminin (1∶40 dilution, L9393; Sigma) in PBS (phosphate buffered saline) containing 0.05% Tween 20 for 30 minutes. The sections were incubated with secondary Alexafluor 350-labeled antibody (1∶130, Invitrogen) to visualize and define the cell borders. Lipid droplets were stained with Oil Red O. Section images were captured and fractional area of intramyocellular lipids (IMCL) was analyzed as previously described [27].

### Muscle Enzyme Assays

Citrate synthase and hydroxylacylCoA dehydrogenase activities were measured spectrophotometrically in *tibialis anterior* muscle samples as previously described [28].

### Muscle Mitochondrial Isolation and Measurements

Skeletal muscle mitochondria were isolated as previously described [29]. Respiration rates in isolated mitochondria (0.1 mg/mL) were determined at 37°C by polarographic oxygen sensors in a two-chamber Oxygraph (OROBOROS® Instruments, Innsbruck, Austria) using pyruvate (5 mM) plus malate (3 mM) or palmitoyl-coenzyme A (CoA) (50 uM) plus carnitine (2 mM) as substrates [29]. Hydrogen peroxide (H_2_O_2_) release from isolated mitochondria fueled by succinate (10 mM) over 30 minutes at 37°C was determined by Amplex Red fluorescence quantification (Invitrogen, Paisley, UK) as described elsewhere [26].

### Mice Insulin Tolerance Test

ITTs were performed in a subgroup of male mice, WT (n = 7−9) and C5L2KO (n = 8−9), after 9 weeks on a low-fat or high-fat diet as described above. Food was removed for 4 h prior to insulin treatment (0.75 U/kg i.p diluted 1∶1,000 in saline, Humilin, Lilly), blood samples were taken 0, 15, 30, 60 and 120 min after insulin injection. Glucose was measured with a glucose meter (LifeScan, Milpitas, CA, USA).

### Human Skeletal Muscle and Plasma Samples

*Vastus lateralis* skeletal muscle samples and plasma were obtained from three studies published previously in older overweight and obese men. The present results represent a subset analysis of these studies based on availability of plasma and skeletal muscle tissue samples; data is included only for samples analysed for C5L2 protein expression [30], [31], [32]. Older obese men with type 2 diabetes (n = 18) and age- and BMI- matched obese male (n = 18) were evaluated for body composition assessment and fasting blood samples and muscle biopsies before and after a 12-week combined aerobic and resistance exercise training intervention and plasma as previously described [31]. Diabetic subjects discontinued anti-diabetes medication 7 days prior to the biopsies and clamp [31]. Fasting plasma glucose, insulin, NEFA and TG measurements, hyperinsulinemic-euglycemic clamps and muscle IMCL were performed as previously described [31]. Plasma ASP was measured as previously described in detail [5]. Due to technical difficulties with sample preparation, plasma ASP was determined in 13 out of 18 obese subjects and 15 out of 18 type 2 diabetic subjects.

### Western Blot

Human skeletal muscle *vastus lateralis* and mouse skeletal muscle *tibialis anterior* samples were homogenized in lysis buffer (1% NP40, 0.5% SDS, 1 mM PMSF in PBS) supplemented with protease inhibitor cocktail (Roche). Equal amounts of protein were loaded onto a 12% polyacrylamide gel and standard SDS-PAGE protocols were followed. For C5L2 protein determination, membranes were blocked with Tris buffer saline (TBS) and Rockland buffer (Rockland Immunochemicals Inc, Gilbertsville, PA, USA) in a 1∶1 ratio for 60 min. GPR77/C5L2 polyclonal antibody (PAB0298, Abnova, Bioconnect, Huissen, Netherlands) antibody was incubated overnight at room temperature in blocking buffer (1∶1,000). After washing, blots were incubated with secondary antibody (goat anti-rabbit conjugated with AlexaFluor800, 1∶1,0000 in blocking buffer, Invitrogen) 60 min, room temperature. Actin content was determined as a loading control (sc-58670, Santa Cruz Biotechnology Inc, Santa Cruz, CA, USA) using appropriate secondary antibody (Invitrogen). Blots were visualized using Odyssey Near Infrared Imager (Licor, Leusden, the Netherlands) OXPHOS protein content in mouse tissue was measured as described elsewhere [26].

### Statistics

Results are expressed as means ± SEM. Statistical differences were analyzed by unpaired Students t-test between WT and KO mice on the same diet. Growth and insulin tolerance curves were analyzed by two-way ANOVA followed by Bonferonni post-hoc test. Subject data was analyzed by Students t-test (paired or unpaired) or repeated measures ANOVA as indicated in the legends. Correlations were analyzed by Pearson linear regression. Significance was set as p<0.05, where NS represents not significant. All graphs and statistical analyses were performed using GraphPad Prism 5.0 (GraphPad Software, San Diego, CA, USA).

## Results

### IMCL Levels

**Figure 1:**
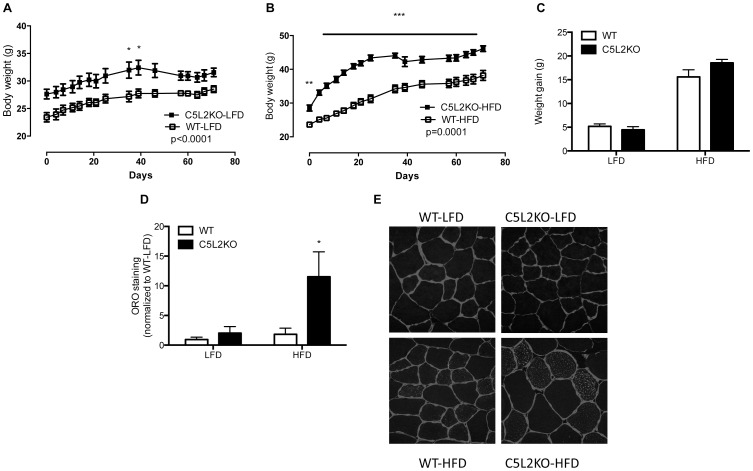
Body weight and IMCL quantification. Growth curves for LFD (A) and HFD (B) WT (open symbols) and C5L2KO (closed symbols) mice over the 10-week diet intervention, n = 6−9 mice. Body weight gain (C) for WT (white bars) and C5L2KO (black bars). Oil Red O staining quantification (D) n = 5 mice per group and representative slides (E) at 40× magnification. Laminin is stained in blue with lipid droplets in red. Data are presented as mean±SEM where *p<0.05, **p<0.01 vs. WT.

Although C5L2KO mice had a higher starting body weight than WT mice (Figure 1A and B), there was no difference in body weight gain (Figure 1C) or adiposity (Table 1) on either diet. Further, as shown in Table 1, there was no difference in epidydimal fat content (% body weight) between C5L2KO and WT. Plasma non-esterified fatty acid levels were similar between WT and C5L2KO mice, with a decrease in fasting plasma triglycerides by 27% in C5L2KO-HFD compared to WT-HFD mice (P<0.01, Table 1). By contrast, skeletal muscle IMCL analysis comparing C5L2KO to WT mice revealed a non-significant twofold increase in IMCL on LFD, with a sixfold significant increase on the HFD (P<0.05, Figure 1D and E).

**Table 1:** Mouse body weight and final plasma values.

Parameters	WT-LFD	C5L2KO-LFD	WT-HFD	C5L2KO-HFD
	N = 6	N = 9	N = 8	N = 9
Epidydimal weight (%/BW)	1.7±0.5	2.1±0.3	5.2±0.9	3.7±0.6
Glucose (mmol/l)	7.84±0.63	8.66±0.64	9.93±1.07	10.63±1.17
Insulin (ng/ml)	1.35±0.17	2.20±0.52	3.23±0.48	8.87±2.70*
NEFA (mmol/l)	0.38±0.06	0.40±0.03	0.46±0.04	0.42±0.05
TG (mmol/l)	1.41±0.14	1.54±0.09	1.82±0.17	1.32±0.07**

Data are presented at mean±SEM and significance was determined by Student’s T-test analysis between WT and C5L2KO on same diet, where *P<0.05 and **P<0.01. Epidydimal weight was determined in a separate cohort.

### Skeletal Muscle Substrate Utilization

**Figure 2:**
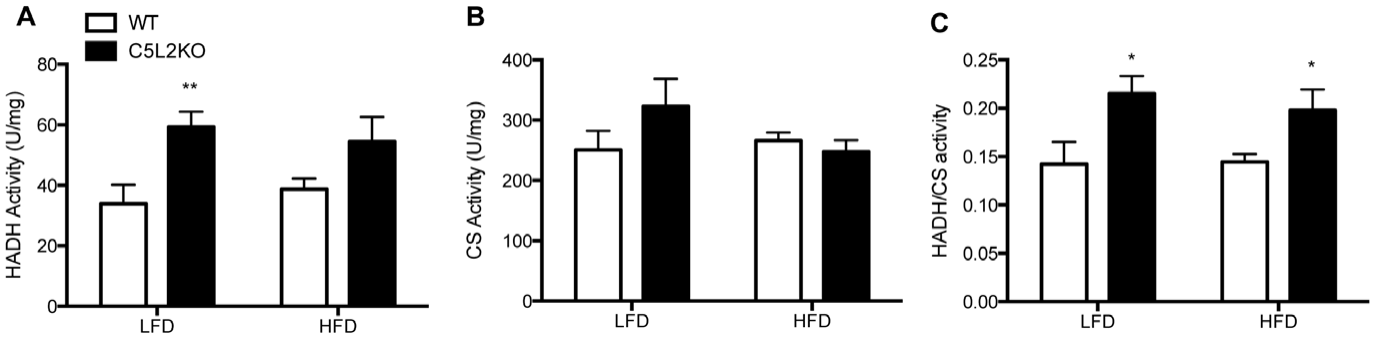
Skeletal muscle enzyme activity. HADH (A), CS (B) activities and HADH/CS ratio (C) for WT (white bars) and C5L2KO (black bars). Data are presented as mean±SEM for n = 6−8 mice per group, where *p<0.05 and **p<0.01 vs WT.

Activity of the beta-oxidation enzyme, hydroxylacyl-CoA dehydrogenase, was significantly higher in C5L2KO-LFD (+75%, p<0.001, Figure 2A) compared to WT-LFD, indicating increased fatty acid oxidation capacity. Meanwhile a slight, but non-significant increase in activity in C5L2KO-HFD (+40%, p = 0.1, Figure 2A) was observed compared to WT-HFD. On the other hand, there was no change in citrate synthase activity between the groups (Figure 2B). However, the ratio of hydroxylacyl-CoA dehydrogenase to citrate synthase (HADH/CS) activity was elevated in both C5L2KO-LFD (+51%, p<0.05) and C5L2KO-HFD (+37, p<0.05) (Figure 2C) mice compared to their WT counterparts, supporting a greater capacity for fatty acid oxidation. In line with the enzyme data, isolated skeletal muscle mitochondria also demonstrated increased FA oxidation (Table 2). Using a lipid-derived substrate, palmityol-CoA (plus carnitine)-supported ADP-stimulated mitochondrial respiration was elevated in C5L2KO mice (state 3 respiration rates LFD: +23%, p = 0.1 and HFD: +23%, p<0.05 compared to WT, Table 2). Maximal respiratory chain respiration (state uncoupled) tended to increase in C5L2KO-HFD (p = 0.08) and there was no difference in leak (state 4) respiration values. By contrast, with a carbohydrate-derived substrate, pyruvate-supported respiration levels were comparable in all groups (Table 2). Meanwhile, hydrogen peroxide production rates, as an indicator of mitochondrial ROS production, were similar between WT and C5L2KO mice for either diet group (Table 2). In addition, no difference was detected in total OXPHOS complex protein count, suggesting similar mitochondrial densities between the study groups (Table 2).

**Table 2:** Mitochondrial respiration rates, ROS production and OXPHOS content.

Respiration rates (pmol/sec/mg)	WT-LFD	C5L2KO-LFD	WT-HFD	C5L2KO-HFD
	N = 6	N = 9	N = 8	N = 9
**Palmitoyl-CoA+Carnitine**				
State 3	1937±170	2389±189	2477±151	3018±115*
State 4	339±26	408±33	400±21	399±33
State Uncoupled	2841±272	3313±164	3307±183	3802±187#
**Pyruvate**				
State 3	6426±613	7544±534	5746±790	6691±319
State 4	410±37	495±43	448±17	450±20
State Uncoupled	10020±1000	12070±1038	9740±623	10140±469
Hydrogen peroxide production (pmol/min/ug)	0.21±0.03	0.24±0.02	0.19±0.01	0.21±0.02
OXPHOS content (arb. units)	2.48±0.37	2.67±0.25	3.10±0.29	3.58±0.36

Data are presented as mean±SEM and significance was determined by Student’s T-test analysis between WT and C5L2KO on same diet, where *p<0.05, #p = 0.08. State 3 (ADP-stimulated respiration) was induced upon the addition of ADP (450 uM), state 4 (leak respiration) was chemically attained with the addition of the ATP-synthase inhibitor oligomycin (1 µg/mL) and the maximally uncoupled state (maximum oxygen flux) was achieved by titrating the chemical uncoupler carbonyl cyanide p-trifluoromethoxyphenylhydrazone (FCCP).

### Whole-body Insulin Sensitivity

**Figure 3:**
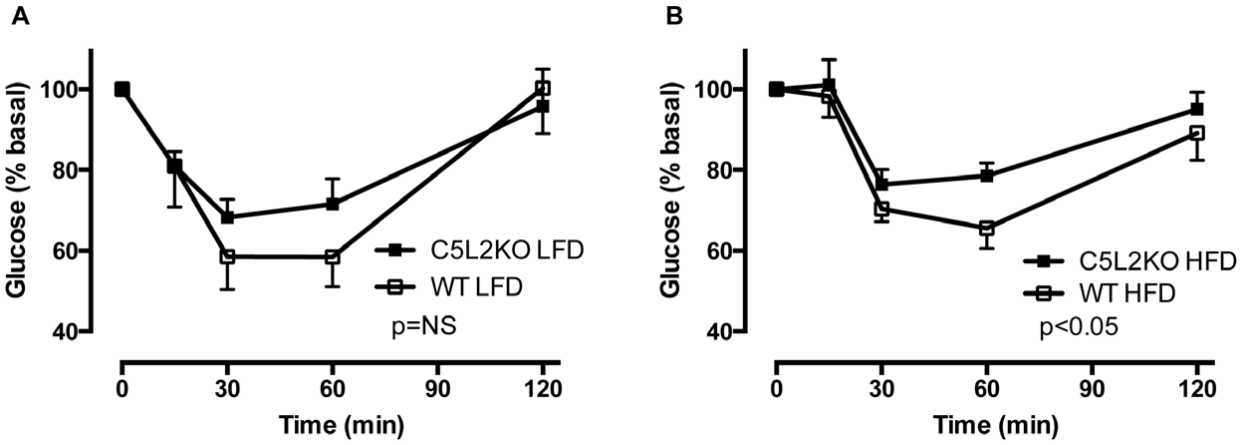
Insulin sensitivity. Glucose response during an insulin tolerance test in LFD (C) and HFD (D) mice for n = 6−9 mice per group. WT (open symbols) and C5L2KO (closed symbols) were fasted for 4 hours before an ip insulin bolus was administered and blood glucose was measured over 2 hours. Data are presented as mean±SEM, two-way ANOVA analysis indicates a significant difference between WT-HFD and C5L2KO-HFD mice (p<0.05).

C5L2KO mice displayed normal fasting glucose and insulin levels on a LFD (Table 1), with no significant difference during the insulin tolerance test (Figure 3A). However, C5L2KO-HFD mice displayed a marked two-fold increase in fasting plasma insulin levels over WT HFD (p<0.05, Table 1). Further, insulin tolerance test results revealed significantly impaired glucose response in C5L2KO HFD mice (p<0.05 by two-way ANOVA, Figure 3B).

### Skeletal Muscle C5L2 Protein Expression in Insulin-resistant Subjects

**Figure 4:**
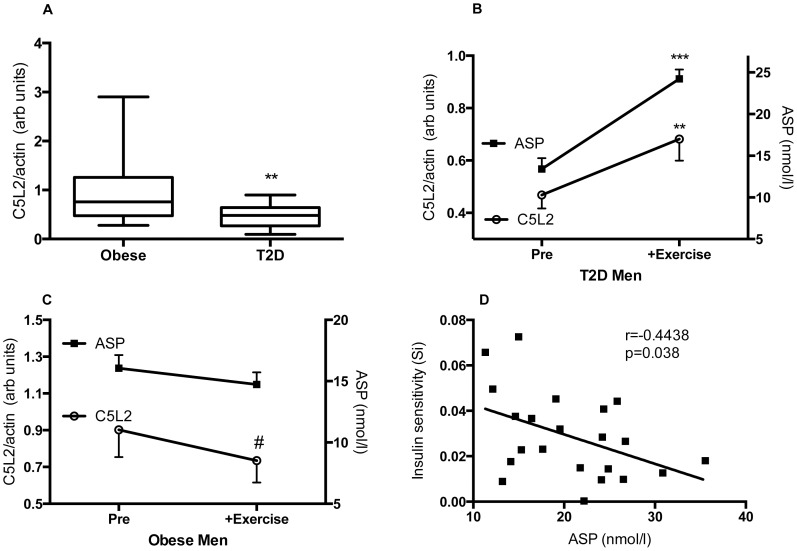
Skeletal muscle C5L2 protein content in humans. C5L2 protein content in obese and type 2 diabetic men (A). Plasma ASP (closed circles) and C5L2 protein levels (open circles) before (Pre) and after (+Exercise) 12-week training regimen in obese (B) and type 2 diabetes male participants (C). Correlation between insulin sensitivity and plasma ASP (D). Data are presented as box and whiskers (with minimum and maximum indicated) (A) was analyzed by unpaired Students t-test and mean±SEM results (B and C) were analyzed by a paired Students t-test, where **p<0.01 and ***p<0.001, #p = 0.08. Plasma ASP and insulin sensitivity were measured in 23 subjects.

Obese type 2 diabetic patients and age- and BMI- matched non-diabetic obese subjects were evaluated from a previous study [31]. For reference, pertinent information on body composition, plasma parameters and IMCL levels are provided for the subjects analyzed (Table 3). As shown in Figure 4A, skeletal muscle C5L2 was significantly lower (−48%, p<0.01) in type 2 diabetic individuals compared to non-diabetic obese men. When all men were taken together, C5L2 protein expression negatively correlated with plasma glucose (r = −0.3953, p = 0.017) and to a lesser extent with IMCL level in type 2 muscle (r = −0.3072, p = 0.07). While there was no difference in fasting ASP levels (Obese: 16.1±1.1 and T2D: 13.4±1.3 pmol/l, p = NS), plasma ASP levels correlated positively with BMI (r = 0.565, p = 0.044), insulin (r = 0.584, p = 0.036), triglycerides (r = 0.586, p = 0.035) and IMCL in type 2 muscle fibers (r = 0.680, p = 0.011), and negatively with C5L2 expression (r = −0.585, p = 0.046) in the obese group only.

**Table 3:** Anthropometric measurements and plasma values for obese and type 2 diabetic men.

	Obese	T2D	Obese	T2D	ANOVA
	pre-training	pre-training	post-training	post-training	
	n = 18	n = 18	n = 18	n = 18	
Age (years)	58.8±0.9	59.3±1.1	–	–	
BMI (kg/m^2^)	30.0±0.9	30.0±0.8	29.7±0.8	29.8±0.2	NS
Glucose (mmol/l)	6.0±0.1	9.0±0.4	5.5±0.1	9.0±0.3	p<0.0001^a^
Insulin (mU/ml)	18.8±2.6	16.4±1.2	16.6±2.3	14.6±0.8	NS
Insulin sensitivity (Si)	0.029±0.005	0.013±0.003	0.034±0.005	0.023±0.003	p = 0.004^a^
NEFA (umol/l)	474.8±24.5	519.7±25.3	463.1±31.0	500.1±34.1	NS
IMCL type 1 fibers (AU)	1.71±0.21	2.18±0.37	1.85±0.41	2.66±0.51	NS
IMCL type 2 fibers (AU)	1.02±0.16	1.10±0.16	0.91±0.18	1.51±0.28	NS

Data are presented at mean±SEM and significance was determined by repeated measures ANOVA where “a” indicates significant subject effects and NS refers to not significant. Insulin sensitivity was measured in 16/18 obese and 15/18 type 2 diabetes participants.

### Effects of Physical Training on Skeletal Muscle C5L2 Protein Expression in Humans

In obese men, a 12-week exercise training intervention improved insulin sensitivity as determined by an hyperinsulinemic-euglycemic clamp [31], with decreases in body fat indices (Table 3). However, while fasting insulin decreased in both groups, plasma glucose decreased only in the non-diabetic obese group (Table 3). In the obese non-type 2 diabetic men, slight but non-significant reductions in skeletal muscle C5L2 protein expression (−17%, p = 0.07) and plasma ASP levels (−18%, p = 0.06, Figure 4B) were observed between pre and post-training states, with a positive correlation between C5L2 and plasma triglycerides post-training (r = 0.6056, p = 0.0077). In type 2 diabetic men, both C5L2 protein expression and plasma ASP levels (Figure 4C) were significantly increased post-training compared to pre-training levels, with a positive correlation between C5L2 expression and BMI post-training (r = 0.4780, p = 0.05). Finally, when both groups were pooled plasma ASP negatively correlated with insulin sensitivity during the hyperinsulinemic-euglycemic clamp (Figure 4D, r = −0.444, p = 0.038).

## Discussion

Ectopic lipid accumulation results from increased FA uptake, upregulated lipid synthesis within the tissue and/or reduced FA oxidation. In the present study we addressed the latter mechanism by evaluating skeletal mitochondrial enzyme activity and respiration in C5L2KO mice. Here we demonstrated that C5L2KO-LFD mice displayed increased fatty acid oxidation capacity, with similar lipid accumulation and insulin sensitivity. C5L2KO-HFD mice exhibited massive skeletal muscle lipid accumulation and altered insulin sensitivity accompanied by upregulated fatty acid oxidation, without changes in mitochondrial density or detrimental ROS production. Furthermore, we determined that in obese men, both insulin sensitivity and exercise impact C5L2 levels.

In the present study we demonstrated that skeletal muscle lipid accumulation in the insulin resistant state was dissociated from muscle fat oxidative capacity. This finding is consistent with results shown by others [33], [34], however, while increased skeletal muscle lipid oxidation during insulin resistance was usually accompanied by increased mitochondrial biogenesis in those studies, our results revealed no change in OXPHOS content or CS activity, both of which are markers of mitochondrial content. Therefore, we propose the lack of C5L2 influences lipid substrate handling rather than mitochondrial density. Further, muscle lipid accumulation is greatly pronounced despite increases in fat oxidative capacity evaluated based on HADH/CS or palmitoyl-CoA supported respiration, suggesting that fatty acid influx to the muscle exceeds the fat oxidative capacity. Furthermore, increased fat storage and utilization in the muscle shown here, coupled to studies by Faraj *et al* in *ex vivo* muscle tissue demonstrating an inhibitory effect of ASP on muscle LPL activity, chylomicron triglyceride hydrolysis and cellular lipid uptake [13], [21], are all consistent with a C5L2 inhibitory role in muscle lipid metabolism, an effect opposite to its role in WAT [9]. In WAT, ASP facilitates postprandial FA trapping by stimulating LPL activity [13], [21]. Interestingly, tissue-specific opposing effects on LPL activities are also recognized for insulin [35], [36]. Thus, in a postprandial situation, insulin stimulates fat uptake in adipose and reduces utilization in muscle via lipoprotein lipase control [10], [21]. The ASP-C5L2 pathway may function similarly, contributing to tissue specific fat partitioning.

C5L2 deficiency not only alters muscle lipid, but also is associated with skeletal muscle insulin resistance. In the present study we demonstrated that C5L2KO-HFD mice displayed elevated fasting plasma insulin levels, coupled with a small but significant impairment in glucose response with high insulin challenge. Both of these results are consisted with those obtained previously in C5L2KO mice on a high-fat high-sucrose diet [25], [24]. suggesting overall that the C5L2KO are more insulin resistant, and need to maintain a higher level of insulin even when fasting to compensate. Meanwhile, in the human study, insulin resistance in the older obese men was associated with decreased C5L2 expression level. Taken together with our previous data that ASP stimulates glucose uptake and glucose transport (Glut3 and Glut4) in differentiated myotubes [20], and the reduction in glucose uptake evident in C5L2KO muscle tissue [25], these results are indicative of a potential role for C5L2 in skeletal muscle glucose metabolism. According to the Randle cycle, when high levels of both fat and glucose substrates are available, the utilization of one substrate inhibits the oxidation of the other [37]. In type 2 diabetes, there is a shift towards increased fatty acid oxidation to the detriment of glucose oxidation leading to impaired glucose uptake [37], consistent with the decreased C5L2 levels. Conversely, exercise increases insulin sensitivity, stimulates glucose utilization, and is associated with the increases in C5L2 levels demonstrated. Of interest, ASP levels increased in response to chronic exercise training in obese subjects. This is contrary to effects seen in an acute exercise intervention, as we have previously reported [3], [7], however the influence of acute vs chronic exercise on ASP is unknown and warrants further investigation.

The studies on C5L2KO mice evaluating lipid metabolism are consistent with previously published studies in C3KO mice, which are deficient in ASP, demonstrating that absence of receptor (C5L2) or ligand (ASP) produces comparable results. Specifically, C3KO (ASP deficient) mice have delayed postprandial triglyceride clearance and increased energy expenditure, indicating re-partitioning of lipid away from adipose tissue storage and towards muscle utilization, which is supported by the *in vivo* and *ex vivo* analyses [38], [39]. C5L2KO mice display a comparable phenotype, although subtler in some aspects on a LFD. Yet, when challenged with a high-fat diet, the C5L2KO phenotype becomes pronounced. In *ex vivo* experiments, adipose tissue from C5L2KO on LF or HF diet demonstrates a reduced capacity for lipid synthesis, as well as the expected absence of ASP stimulation [24], [40]. Nonetheless, it should be noted that, in the present study, the impact of increased body weight on skeletal muscle fat accumulation in C5L2KO-HFD mice cannot be ignored.

As the mouse C5L2KO model is a whole-body gene deletion, one limitation is that we cannot determine if *in vivo* muscle effects are due to muscle C5L2 absence, or consequent to absence of C5L2 in other tissues (adipose or elsewhere). We cannot dismiss the possibility that the drive in increased fatty acid oxidation and lipid accumulation in muscle may be attributed to simple overflow of lipids derived from reduced storage in WAT or related to increased body weight. Further, while we did show that the fatty acid transporter, CD36, expression was unaltered in C5LKO mice on a high-fat high-sucrose diet [24], [25], we cannot assume this to be true on a diet high in fat only. Similarly, in the human studies, while changes in C5L2 may be reflective of direct effects on muscle, the impact of other tissue influences cannot be ruled out. Finally, it cannot be disregarded that C5L2 is also a receptor for C5a and C5adesArg. Thus the absence of C5a/C5adesArg interaction with C5L2, or an enhanced interaction of C5a/C5adesArg with its alternate receptor, C5aR, could potentially contribute to the present phenotype, although no role for C5a in lipid/glucose metabolism has yet been identified.

Overall, in an obese state, such as mice on a HFD, muscle fatty acid oxidation induced by C5L2KO is associated with insulin resistance and accrued lipid content. Similarly, in obese men, changes in muscle function induced by an insulin resistant state or exercise training alter C5L2 expression levels. Altogether, these data suggest that insulin sensitivity may be permissive for coupling of C5L2 levels to lipid accumulation and utilization. What signals might be directly regulating these increases or decreases in muscle C5L2? While this remains unknown, in adipocytes insulin and insulin-sensitizers (TZD) upregulate both C5L2 mRNA and cell-surface protein expression [41], while ASP augments glucose-stimulated insulin secretion through a direct action on the islet beta cells [42]. By contrast, TNF-alpha and fatty acids downregulate C5L2 in adipocytes [41], [43]. All of these could potentially play a role in C5L2 changes in humans. This raises the question whether ASP-C5L2 influences insulin sensitivity directly or if insulin-sensitivity regulates C5L2 expression; further studies are required to determine this relationship.

### Ethical Statement

The study involving human participants was approved by the Maastricht University’s institutional medical ethics committee and complied with the principles of the Declaration of Helsinki. All participants were informed of the risks involved in the study and gave their written informed consent.

All animal protocols were approved by the Maastricht University and Laval University and were conducted in accordance with its guidelines, and all efforts were made to minimize suffering.

Mice were housed 2 per cage in a sterile animal facility with a fade in/fade out 12 h light/dark cycle. The cage also contained enrichment toys such cotton bating and plastic tubes. Prior to dissection, the mice were first sedated (CO2 and O2 (67∶33%) sedation), followed by immediate decapitation, and tissues were then collected.
